# Gleanings from Our Exchanges

**Published:** 1873-03-15

**Authors:** 


### Gleanings from Our Exchanges.

### TRANSLATIONS FROM THE FRENCH.

BY DR. BOSWORTH, CHICAGO.

The *Gazette Medicale de Paris* (Jan. 25, 1873) says:

Dr. Henry Roger, member of the Academy of Medicine, Physician of the Children's Hospital, Adjunct Professor of the Medical Faculty of Paris, has published a remarkable work on "Diseases of Children."

From the very interesting chapter on "The Temperature in Diseases of Children," we find the following, quite worthy of translation:

The temperature of a new-born child is a very little higher than that of the mother. From the first to the seventh day the *mean* temperature is 37°.08*. At this moment the number of inspirations has very little influence on the degree of heat, while the acceleration of the pulse, as well as the force of the child, cause the thermometer to rise. The temperature of girls he has always found lower than that of boys; lower also during wakefulness than during sleep.

From one to six years of age he found the pulse of 102, and the temperature 37°.11; from six to thirteen years, pulse 77, temperature 37°.31. Neither exercise nor digestion augments heat.

As to the region for observing the temperature of children, he prefers the axilla to the rectum; although in the latter region the thermometer rises higher, nevertheless the axilla is much more convenient, and has nearly the same temperature as the abdominal and thoracic cavities. In reality, there is almost no difference between the temperature of the axilla and rectum in young children.

After these general remarks he then reviews the different diseases in which the temperature increases.

In *ephemeral* fever the thermometer rises sometimes as high as 40°. In intermittent fever, during the algid period (after Garvarret and Roger), there is an increase of heat; but the patient is really cold, on account of the unequal distribution of the animal heat, the cutaneous surface being cold to the benefit of the internal heat, which is augmented. During the interval of the attacks, and in the second stage of the disease (*stare de chaleur*), the thermometer rises, but less than with adults. The pulse and the respiration decrease during the second stage.

* The degrees here referred to are the French centigrade.

In hectic fever the thermometer did not rise above 39°.2, although the pulse was very frequent.

Typhoid fever, after Mr. R., proves the most emphatically that the source of heat in man is not only in the respiration and circulation. Out of twenty-three cases, he saw, in fourteen of them, the thermometer rise to 40°, and even above, the highest being 41° in the morning. If only the grave cases are taken, he found an average temperature of 40°.14. The mean temperature of the whole twenty-three cases was 39°.77. The duration of this elevated temperature is often quite long. Generally, where the heat is excessive, the cases are more dangerous; nevertheless, he cites one case of a child, twelve years old, having on the sixth day the thermometer at 41°, pulse 92, yet it recovered in one month. Another child, thirteen years old, having on the seventh day a temperature of 40°, was well in three weeks.

The author has found that the development of heat at the very beginning of typhoid fever, varies according to the way in which it begins; if this disease commences suddenly, with a high fever, the temperature will be directly very high; if, on the contrary, as is more frequently the case, it begins by malaise, shivering, want of appetite, etc., symptoms which last some days without any decided character, the heat, although already high, is much less intense than it will be at a later period. The thermometer increases and decreases with the disease itself. During convalescence the temperature sometimes descends below the natural, ordinary degree, although the amelioration of the pulse continues.

In the adynamic form of this fever, the temperature is relatively but little elevated; here he observed, as a maximum, 39°.05. He relates one case where the maximum had been 38°.75, but, on the day of death, it was 40°; another case, where on the day of death it was 36°.75.

In the inflammatory form, the maximum is higher. As a general rule, the pulse is slow, compared to the elevation of temperature; nevertheless, he found the pulse to increase in frequency while the temperature was augmenting, and to decrease as the thermometer fell. In the greater number of cases he found a connection between the acceleration of the respiration and the elevation of temperature. The principal points for the diagnosis of typhoid fever are the following: As a general rule, in the first stage of this disease (swelling and softening of Peyer's patches), the thermometer augments from 1-2° to 1° daily, with an exacerbation in the evening. Wunderlich and Griesinger concluded that we have not a case of typhoid fever if the thermometer indicates, on the second evening, 40°; or, if on the fourth evening, it does not reach 39°.5; or, if from the eighth to the eleventh day, it does not reach 39°.5. Mr. R. says we must occasionally make an exception to this rule for children, as he has seen one child die with only 38°.4 on the sixth day; another, where it did not go above 39°.4, and 37°.8 from the eighth to the eleventh day. After this author it is the only disease where a high temperature sometimes coincides with a pulse of moderate frequency.

In a case of variola, we might suspect, before the eruption, typhoid fever, if the thermometer did not reach, by the second or third day, 40°. As soon as the eruption of variola appears, the temperature suddenly falls. A very high degree of the thermometer during the first few days announces a pneumonia.

Mr. R. declares it to be impossible, by the aid of the thermometer, to diagnose typhoid fever from the exanthema, from rheumatism, etc. As to prognosis, high temperature at the end of the first week indicates a very grave case. 40° and 41°, continuing several days, announce a fatal termination. It is here impossible to indicate critical days, as with adults, the disease being shorter with children on account of its gravity with them. Death is also to be feared when we have a very low temperature caused by anemia. In cases of high temperature, Mr. R strongly praises cold baths. Among the eruptive diseases, the thermometer rises the highest in scarlatina, 39°.4; in variola, 38°.7. In roseola it only rises to 38°.5.

In erysipelas, he found the thermometer at its maximum at the moment of the appearance of the exathema. The places attacked have a temperature superior to the regions unattacked (40°, 40°.5), never superior, however, to that of the rectum or axilla.

In rheumatism and gout, there is a slight increase at the diseased points.

In stomatis, the increase is slight (38° in the mouth, 37°.75 in the axilla). Little elevation also in enteritis; in the latter there is no constant relation between the pulse and the temperature. As a diagnostic sign between typhoid fever and enteritis, tle latter has a lower temperature.

The wide range of the thermometer in meningitis is remarkable, being from 35° to 42°.5. The average temperature in this disease, 38°.5, 39°.5. Near the close of this disease the temperature is the highest. Sometimes the elevation is sudden, as much as 5 1-2° in one day. Such great variations of temperature are not explained by age, by the seat of the disease, by the intensity, or by the form acute or granuleuse. The faster the progress of the disease, the greater is the elevation; the decrease in the rapidity of the inspirations coincides with the falling of the thermometer. It is only in meningitis that we have a notable lowering of the temperature in the middle of the disease. In face of general violent symptoms, if the thermometer surpasses 40° or 41°, it is typhoid fever; if it is below 39°, it is very probably meningitis. It is also probably the latter when the thermometer suddenly changes; also, if the pulse is very changeable.

In encephalitis, cerebral softening, the thermometer raises but little; the pulse is rapid; the respiration accelerated.

In endocarditis, pericarditis, croup, there is but slight elevation of temperature.

The same in bronchitis (37°.7 to 39°), and it is this feature that distinguishes pneumonia from bronchitis, accompanied by dyspnœa and intense fever. The grippe may give a temperature of 40°.6.

In acute pleurisy, he found the maximum to be 40°. Sometimes there is a slight difference in the temperature of the two sides of the thorax. The pulse and dyspnœa diminished at the same time with the decrease in temperature.

In pure infantile pneumonia, there is an elevation of temperature for seven or eight days, then a sudden decrease. In secondary broncho-pneumonia, the elevation continues longer, but with remissions and exacerbations; these latter corresponding to new inflammatory attacks. Decrease of temperature at the end of the disease exists, even if the case is mortal. Mr. R. Law, in three cases, on the day of death, the thermometer at 39°.38. If recovery is to take place, the decrease of temperature is greater. The extent, seat, and gravity of the disease, have but little influence on the thermometer. It is the age that limits the increase of the heat. When the temperature rises, the pulse is accelerated; the *vice versa* is not so often true. The relation between the respiration and the temperature is less marked than between the degree of heat and the number of pulsations. Mr. R. remarks that in no disease of children is the relation between the functions of respiration, heat, and circulation more pronounced than in inflammation of the pulmonary tissue; in none do we find a simultaneous, higher average, the temperature being 39°.97, pulse 133, and 52 respirations.

With children, the diagnosis between lobular pneumonia and bronchitis is difficult; but in the former we have sometimes 41.°27, while in the latter it seldom surpasses 38°. The diagnosis between pleurisy and pneumonia is based on the sudden elevation of the thermometer, on the latter, to 40°, 40°.5, or 41°. As to typhoid fever, it is distinguished from pneumonia by the temperature of 40° being reached in the latter in the first two days, and by the acceleration of the respiration; the mean respiration in pneumonia being 52, in typhoid fever 37.

As to treatment, Zeemsen has seen cold compresses applied on the thorax produce a falling of 1° to 3°, of short duration only, and a reduction of 24 pulsations. Bleeding produces a still less durable decrease. Tartrate of antimony causes the thermometer to fall from 40° to 36°.4 on the fourth day; the pulse from 132 to 84. Digitalis, on the fifth day, caused a reduction from 40° to 38°.7; but in this case, on the following day, it was again 40°. On the seventh day, defervescence.

Mr. Roger now passes to diseases which are characterized by a stationary temperature.

Tubercles, according to his observations, do not produce any augmentation, except by their complications. He rejects the doctrine of Wunderlick and Sidney Ringer, who pretend to diagnose tubercles by the aid of the thermometer. At the beginning, there is a slight increase of temperature; then comes the hectic fever, with oscillations of the thermometer—the exacerbations of the evening being more characteristic in this disease than in any other. The temperature is higher in thoracic tubercles than in those of the abdomen—in adults than in children (Andral). In two-thirds of the cases with children there is accord between the respiration, temperature, and the pulse. In cerebral tubercles, the exacerbations are remarkable. When there are new inflammatory attacks around the tubercles, Mr. R. calls attention to the fact that the temperature is little changed, although the nervous system, to which a great influence or *calorification* is generally admitted, is so severely attacked. In chorea and epilepsy there is no elevation; although convulsions augment the temperature after Becquerel and Breschet.

Now come the diseases in which the temperature falls.

There is a decrease of 17°, sometimes, in paralysis of the limbs. If only one side is taken, it may be that the temperature is less there than in the unaffected side.

In cholera, there is local and general decrease of heat; and the greater the decrease the greater the gravity; the temperature diminishing 4 °or 5° in the mouth, or 2° or 3° in the axilla, indicates danger. Sometimes, here, a quick pulse coincides with a low temperature.

In œdema of new-born children, the thermometer falls sometimes from 33° to2 2° (in the axilla, after Roger; in the rectum, after Parrot). At the same time, there is a complication which has unjustly been called a pneumonia, and which does not prevent the great decrease of heat. This complication is in reality an apoplexy, a congestion, and not an inflammation. Mr. R. has seen one of these cases of œdema, with the themometer at 32°.5 and 33°, recover. There is here a close relation between the pulse and the respiration. Sometimes the pulse was 60, and the respiration 14. Mr. R. attributes this Algid œdema of infants to two causes: First, to congenital or acquired weakness, which is found with children born before the full term, or in a state of inanition. Second, to the low temperature of the air—this disease being more frequent in winter. The author says there is here no Bright's disease; the kidneys are healthy. He recommends that the little patients be warmed and fed. There is some chance of saving the child, if the temperature, at first, has not fallen more than 4° or 5°.

Alcohol and Renal Disease.—It seems probable from discussions which have taken place lately in the *London Lancet* and other British journals, that the profession has been in error in regarding the immoderate use of alcoholic drink as tending to produce kidney disease. Dr. Dickinson has been at considerable pains to collect statistical information on this point ; he has also looked into the whole subject very closely, and has arrived at the conclusion that drinking habits are not, on the whole, great contributors to the general mortality from kidney disease. This view is diametrically opposed to a wide-spread medical belief, and one which is sure to meet with considerable opposition. It is a well-known physiological fact that only a small quantity of the alcohol taken into the system is eliminated by the kidneys. It cannot be, therefore, that the cells of the uriniferous tubes are overtaxed in the elimination of alcohol. Besides, it has never been proved, either by clinical observation or by statistical evidence, that there is any decided tendency of alcohol excess to produce kidney disease. The subject is a very important one, and requires careful investigation.—*Canada Lancet.*

Colds—Stop Them.—Dr. Dobell, in his recent work on winter cough, says, in emphatic italics, "*colds can be stopped without lying in bed, staying at home, or in any way interfering with business.*" He says that his plan, if "begun directly the first signs of catarrh show themselves in the nose, eyes, throat or chest, . . . is almost infallible," but "will not answer if the cold has become thoroughly established."

The plan is as follows :

"1. Give five grains of sesquicarbonate of ammonia, and five minims of liquor morphia in an ounce of almond emulsion every three hours. 2. At night, give ℥iss. of liquor ammonia acetis in a tumbler of cold water, after the patient has got into bed and been covered up with several extra blankets ; cold water to be drunk freely during the night should the patient be thirsty. 3. In the morning, the extra blankets should be removed, so as to allow the skin to cool down before getting up. 4. Let him get up as usual, and take his usual diet, but continue the ammonia and morphia mixture every four hours. 5. At bed-time, the second night, give a colocynth pill. No more than twelve doses of the mixture from first to last need be taken, as a rule ; but, should the catarrh seem disposed to come back after leaving off the medicine for a day, another six doses may be taken, and another pill. During the treatment the patient should live a little better than usual, and on leaving it off should take an extra glass of wine for day or two."

Dr. Dobell says his patients call this the "magic mixture."—*Boston Med. & Surg. Journal.*

McIntosh on Dysmenorrhœa and its Treatment with Sulphate of Quinia and Extract of Stramonium Seeds.—The results of an experience with each of these drugs, used separately, led Dr. McIntosh (*American Quarterly Journal of Medical Science*, Jan. 1873) to unite them in the following proportions, varied according to the requirements of each individual case: "Give a pill consisting of one-fourth to one-third grain ext. daturæ stramon. sem. ; one-half to three grains sulph. quinia ; one-fourth to one-half grain opii ; one to two grains camphor, three times a day for five days, beginning three days before the catamenial discharge, and continuing for two days after its inception. The same treatment is to be commenced just previously to the next monthly period; and usually from four to eight repetitions, where there is no mechanical obstruction, will secure a regular, painless monthly flow." Latterly Dr. McIntosh has added powdered ipecacuanha to the above pill, and, as he states, "with benefit." "With the foregoing treatment should always be combined such emmenagogue and furruginous medicines as an anemic or other condition may require, while special directions should be given to procure a daily action of the bowels. A careful avoidance must be observed of exposure to cold or wet, and great care in keeping the feet warm, and a good circulation in the lower extremities generally.—*Medical Record.*

Chauvin on Cæsarean Section : Recovery.—Dr. Chauvin (*Journal de Med. l' Ouest*, 4me trimestre, 1872) gives the details of a case in a rickety patient, aged twenty-six, with a very contracted pelvis antero-posteriorly (3½ centimetres=1.37 inch). No cephalotribe was at hand, though, had it been, he would gladly have employed it, as, from the cessation of the movements of the fœtus and absence of the heart-sounds, its death was feared. Hysterotomy was performed in the usual way—a verv large dead fœtus being extracted. Rigors and fevers ensued. Mercurial inunctions were employed. Convalescence was complete by the twenty-first day. Dr. Chauvin thinks that the history of this case should encourage country practitioners to resort to the operation under similar circumstances.—*Medical Record.*

Cohnstein on Determination of the Life or Death of a Fœtus.—Dr. Cohnstein (in *Arch. fur Gynak.*, vol. iv. 3d part, 1872) states that the information whether the fœtus is living or dead during pregnancy, but especially during parturition, is often of the greatest importance ; and where hearing the fœtal heart and feeling the fœtal movements fail or are uncertain, ascertaining the temperature *in utero* will often very materially assist if not decide us in determining the question. It is a fact that the temperature of the fœtus *in utero* is higher than the maternal temperature ; and experience proves that the careful introduction of the thermometer into the uterine cavity between the membranes and the wall of the uterus is unattended by harm. We have thus a ready mode of settling the question when it is otherwise doubtful.—*Medical Record.*

The degree of Doctor of Medicine was conferred on twenty candidates at the close of the recent spring examination at Harvard Medical School.

Dr. Tilt was recently elected President of the Obstetrical Society of London; and Sir William Jenner has been chosen President of the Pathological Society.

THE SEWERAGE QUESTION.—This is the most prominent sanitary question now under examination in England, and many costly experiments are being tried by private companies and municipalities in the endeavor to turn sewerage to profitable account, without danger to public health. We have a large number of printed documents relating to this important subject, but refer the reader for its general discussion to the fourth report of the State Board of Health of Massachusetts, now in press.

The following sentence, which we quote from a recent writer in the London *Medical Times & Gazette*, well and briefly expresses the object of all efforts in this direction :

"As life involves the conversion of food into sewerage, so science must convert sewerage into food, and deal with it so as to make the earth fertile, the air pure, and the water sweet."—*Boston Med. & Surg. Journal.*

